# Endoplasmic reticulum Ca^2+^-homeostasis is altered in small and non-small cell lung cancer cell lines

**DOI:** 10.1186/1756-9966-28-25

**Published:** 2009-02-24

**Authors:** Albrecht Bergner, Julia Kellner, Amanda Tufman, Rudolf M Huber

**Affiliations:** 1 Division of Respiratory Medicine, Medizinische Klinik-Innenstadt, Ludwig-Maximilians-University, Munich, Germany

## Abstract

**Background:**

Knowledge of differences in the cellular physiology of malignant and non-malignant cells is a prerequisite for the development of cancer treatments that effectively kill cancer without damaging normal cells. Calcium is a ubiquitous signal molecule that is involved in the control of proliferation and apoptosis. We aimed to investigate if the endoplasmic reticulum (ER) Ca^2+^-homeostasis is different in lung cancer and normal human bronchial epithelial (NHBE) cells.

**Methods:**

The intracellular Ca^2+^-signaling was investigated using fluorescence microscopy and the expression of Ca^2+^-regulating proteins was assessed using Western Blot analysis.

**Results:**

In a Small Cell Lung Cancer (H1339) and an Adeno Carcinoma Lung Cancer (HCC) cell line but not in a Squamous Cell Lung Cancer (EPLC) and a Large Cell Lung Cancer (LCLC) cell line the ER Ca^2+^-content was reduced compared to NHBE. The reduced Ca^2+^-content correlated with a reduced expression of SERCA 2 pumping calcium into the ER, an increased expression of IP_3_R releasing calcium from the ER, and a reduced expression of calreticulin buffering calcium within the ER. Lowering the ER Ca^2+^-content with CPA led to increased proliferation NHBE and lung cancer cells.

**Conclusion:**

The significant differences in Ca^2+^-homeostasis between lung cancer and NHBE cells could represent a new target for cancer treatments.

## Background

Lung cancer is the leading cause of cancer death in the industrial nations [1]. Despite recent advances, therapeutic regimens support quality of life but frequently fail to increase long term survival. One of the main reasons for the failure of therapeutic regimens is the fact that cancer cells originate from normal cells and therefore possess similar characteristics. This means that anti-cancer therapies inevitably affect the normal cell population and these side effects often hinder more effective treatments. Thus, knowledge of the differences in the cellular physiology between malignant and non-malignant cells is crucial for the development of more successful treatments.

**Figure 1:**
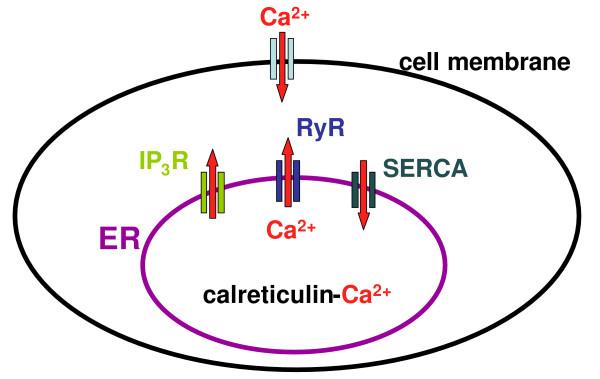
**Increase in the cytoplasmic Ca^2+^-concentration can be due to Ca^2+^-influx from the extracellular space or due to Ca^2+^-release from the endoplasmic reticulum (ER)**. The equilibrium of the ER Ca^2+^-content is maintained by sarcoplasmic/endoplasmic reticulum Ca^2+^-ATPases (SERCA) pumping calcium into the ER and inositol-1,4,5-phosphate- (IP_3_R) and ryanodine-receptors (RYR) releasing calcium out of the ER. Within the ER, calcium is mainly buffered by calreticulin.

Calcium is a ubiquitous signal molecule that is involved in almost all cellular pathways [2,3]. Elevation of the cytoplasmic Ca^2+^-concentration ([Ca^2+^]_c_) can result either from Ca^2+^-influx from the extracellular space or from Ca^2+^-release from internal Ca^2+^-stores, primarily the ER. Proteins involved in the Ca^2+^-release from the ER are the inositol-1,4,5-trisphosphate receptor (IP_3_R) and the ryanodine receptor (RyR) (Figure 1). Sarco/endoplasmic reticulum Ca^2+^-ATPases (SERCA) force calcium against the concentration gradient from the cytoplasm into the ER. Within the ER, calcium is buffered by calreticulin [2,3]. Calcium is particularly important for the regulation of proliferation and apoptosis and the imbalance of cell growth and cell death finally leads to cancer. The aim of this study was therefore to evaluate whether the ER Ca^2+^-homeostasis is altered in lung cancer cell lines compared to normal bronchial epithelium.

## Methods

### Materials

Cell culture reagents were obtained from Life Technologies (Eggenstein, Germany). Other reagents were bought from Sigma-Aldrich (Deisenhofen, Germany) unless stated otherwise. The human lung carcinoma cell lines H1339 (Small Cell Lung Carcinoma), DMI 53 pI (Small Cell Lung Carcinoma), LCLC-103H (Large Cell Lung Carcinoma), EPLC 272 (Squamous Cell Lung Carcinoma), EPLC M1 (Squamous Cell Lung Carcinoma) and HCC (Adeno-Carcinoma) were purchased from the German Collection of Microorganisms and Cell Cultures (DSMZ, Braunschweig, Germany). Primary normal human bronchial epithelial cells (NHBE) were purchased from Lonza (Walkersville, MD, USA).

### Ca^2+^-imaging

For quantification of changes in the [Ca^2+^]_c_, cells were loaded for 30 min at 37°C with the calcium indicator dye Fluor-4 AM (10 μM, Molecular Probes, Eugene, OR) in supplemented Hanks Balanced Salt Solution (sHBSS) containing 0.2% Pluronic (Pluronic F-127, Calbiochem, La Jolla, CA). After loading, the cells were incubated for at least 30 min in sHBSS to allow for complete dye deesterification and examined with a fluorescence microscope (Axiovert 200 M, Carl Zeiss, Jena, Germany). Images were recorded in time lapse (1 frame/sec) using a digital CCD camera (AxioCam MRm, Carl Zeiss Vision, Munich, Germany). For each image, regions of interest (ROIs) were defined in single cells, and the average fluorescence intensity of each ROI was measured. Final fluorescence values were expressed as a fluorescence ratio (F/F_o_) normalized to the initial fluorescence (F_o_). Each analysis was performed using custom written macros in the image analysis software "Scion".

### Western Blot analysis

Protein expression was determined by immunoblotting with protein extracts prepared with the Compartmental Protein Extraction Kit according to the manufacturer's instructions (Chemicon International, Hampshire, United Kingdom). EGFR was used as control for plasma membrane contamination, which was found to be low with no differences between cell types. Protein concentrations of the extracts were quantified using the Bradford Protein Assay Kit (Bio-Rad, Munich, Germany). Proteins were separated by SDS polyacrylamide gel electrophoresis (SDS-PAGE) on a 6% separating and 4% stacking gel (for SERCA 2), on a 4% separating and 3% stacking gel (for IP_3_R and RyR) and on a 10% separating and 4% stacking gel (for calreticulin) and transferred to nitrocellulose membranes (Hybond ECL Membrane, Amersham Biosciences, UK). After blocking for 2 hours in a 5% solution of non-fat dried milk/TBST (TBS with 0.05% Tween 20), the membranes were incubated overnight at 4°C with specific antibodies (SERCA 2 Abcam 1:1000, Mouse anti-Ryanodine Receptor Chemicon 1:500, Mouse anti-IP3 Receptor Chemicon 1:500, anti-Calreticulin antibody Sigma-Aldrich 1:4000, Beta Actin Antibody (HRP) Loading control Abcam 1:5000, SERCA1 ATPase antibody [VE121G9] Abcam 1:500, SERCA3 ATPase antibody Abcam 1:200). Sheep anti-mouse IgG horseradish peroxidase linked whole antibodies (Amersham Biosciences, UK, 1:1500) were used as secondary antibodies. β-actin served as a loading control. Antibody complexes were visualized using Hyperfilm ECL chemiluminescence (Amersham Biosciences, UK) and evaluated using the "Image-J" analysis software.

### Statistics

One-way ANOVA or "ANOVA repeated measurements" (combined with pairwise multiple comparisons) were performed using the "Sigma Stat" software (Jandel Scientific, Chicago, IL). A *P *value of less than 0.05 was considered statistically significant.

## Results

**Figure 2:**
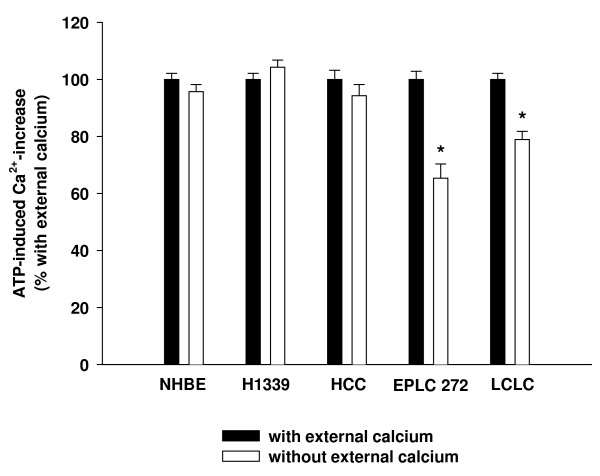
**Cells were exposed to 1 mM ATP in the presence and the absence of extracellular calcium**. The resulting increase in the cytoplasmic Ca^2+^-concentration was quantified using fluorescence microscopy. For each cell line, the Ca^2+^-increase with external calcium was set to 100% (black columns) and the Ca^2+^-increase without external calcium (white columns) was expressed as percent of the increase with external calcium. In normal bronchial epithelial (NHBE), Small Cell Lung Cancer (H1339) and Adeno-Carcinoma (HCC) cells, the ATP-induced Ca^2+^-increase was independent of the presence of extracellular calcium suggesting a minor role for Ca^2+^-influx. In Squamous Cell Lung Carcinoma (EPLC) and Large Cell Lung Carcinoma (LCLC) cells, the ATP-induced Ca^2+^-increase was lower in the absence of extracellular calcium (n = 42 – 162 cells, * = *P *< 0.001 versus "with external calcium").

To investigate the role of Ca^2+^-influx in Ca^2+^-homeostasis in lung cancer cells, NHBE (normal human bronchial epithelial), H1339 (small cell lung carcinoma), HCC (adeno carcinoma), EPLC 272 (squamous cell carcinoma) and LCLC (large cell lung carcinoma) cells were exposed to 1 mM ATP in the presence and the absence of extracellular calcium (PBS containing no calcium but 0.02% EGTA). The resulting increase in the [Ca^2+^]_c _was quantified using fluorescence microscopy. Baseline fluorescence values were similar in all cell lines (data not shown). In NHBE, H1339 and HCC cells, the ATP-induced Ca^2+^-increase was comparable with and without external calcium suggesting an insignificant role for Ca^2+^-influx (Figure 2). In EPLC 272 and LCLC cells, the ATP-induced Ca^2+^-increase was lower in the absence of extracellular calcium.

**Figure 3:**
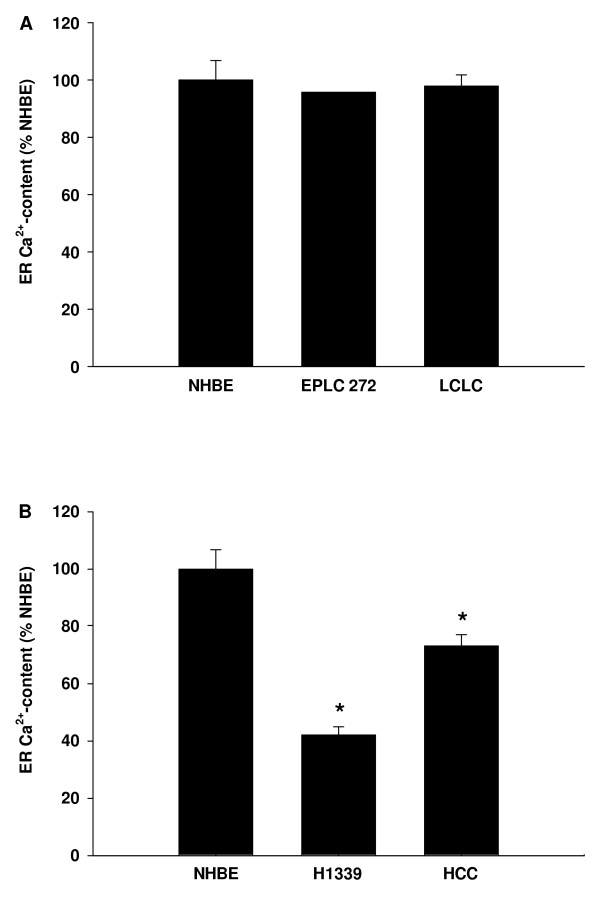
**SERCA were inhibited using 1 μM cyclopiazonic acid leading to a net Ca^2+^-efflux out of the ER**. The resulting increase in [Ca^2+^]_c _was used as an estimate of the [Ca^2+^]_ER _and expressed as percentage of NHBE. **(A) **In EPLC and LCLC cells in which Ca^2+^-influx contributed to the ATP-induced Ca^2+^-increase the [Ca^2+^]_ER _was equal to the [Ca^2+^]_ER _of NHBE. **(B) **In H1339 and HCC cells in which the Ca^2+^-influx did not contribute to the ATP-induced Ca^2+^-increase the ER Ca^2+^-content was lower than in NHBE cells (n = 50 – 153 cells, * = *P *< 0.001 versus all other groups).

Direct measurement of the ER Ca^2+^-concentration ([Ca^2+^]_ER_) is not reliably feasible. Therefore, we used an indirect approach. SERCA were inhibited using 1 μM cyclopiazonic acid (CPA) leading to a net Ca^2+^-efflux out of the ER. The resulting increase in [Ca^2+^]_c _was used as an estimate of the [Ca^2+^]_ER _[4]. In the lung cancer cell lines in which Ca^2+^-influx contributed to the ATP-induced Ca^2+^-increase (EPLC 272 and LCLC) the [Ca^2+^]_ER _was equal to the [Ca^2+^]_ER _in NHBE (Figure 3A). In those lung cancer cell lines in which the Ca^2+^-influx did not contribute to the ATP-induced Ca^2+^-increase (H1339 and HCC) [Ca^2+^]_ER _was lower than in NHBE. The SCLC line H1339 showed the lowest [Ca^2+^]_ER _(Figure 3B).

**Figure 4:**
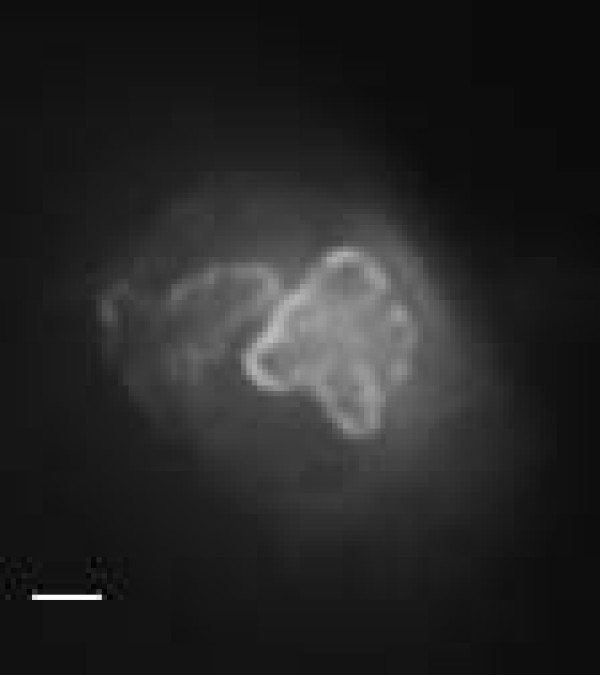
**Immunohistochemical staining of SERCA 2 in a H1339 cell**. Note the ER-typical pattern of the staining as SERCA is an ER-trans-membrane protein. Bar = 2 μm.

**Figure 5:**
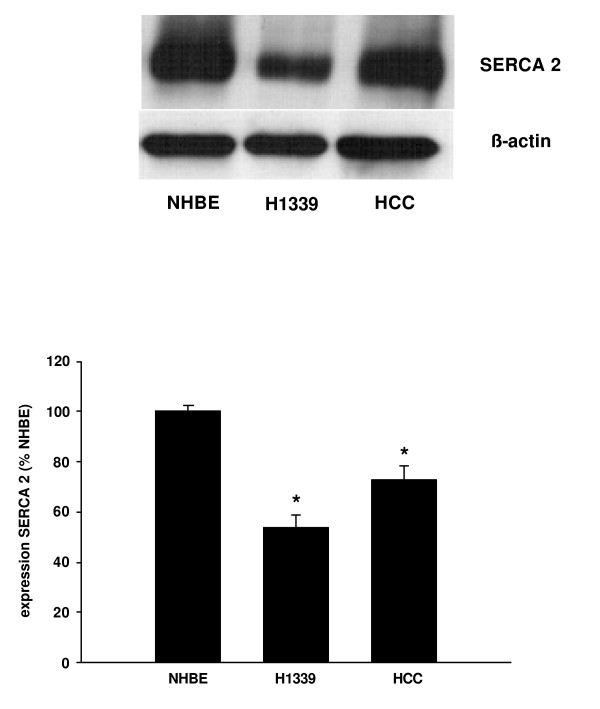
**The expression of SERCA 2 was analyzed in NHBE, H1339, and HCC cells using Western Blot analysis and expressed as percentage of the SERCA 2 expression in NHBE cells**. In H1339 and HCC cells, the expression of SERCA 2 was found to be reduced with H1339 showing the weakest expression (n = 3, * = *P *< 0.01 versus all other groups).

Next, we investigated the expression of the proteins that regulate the [Ca^2+^]_ER_. SERCA pump calcium from the cytoplasm into the ER. Three isoforms of SERCA of have been identified so far and, of these, SERCA 2 has been reported to be the most widely expressed [5]. Analyzing the SERCA expression using Western Blot analysis, we found the isoforms SERCA 1 and 3 to be very weakly expressed (data not shown), while SERCA 2 showed strong expression as confirmed by immuno-fluorescence (Figure 4). Comparing the expression of SERCA 2 in NHBE, H1339, and HCC cells, we found lower levels of expression in the lung cancer cells with expression in H1339 cells being the lowest (Figure 5).

**Figure 6:**
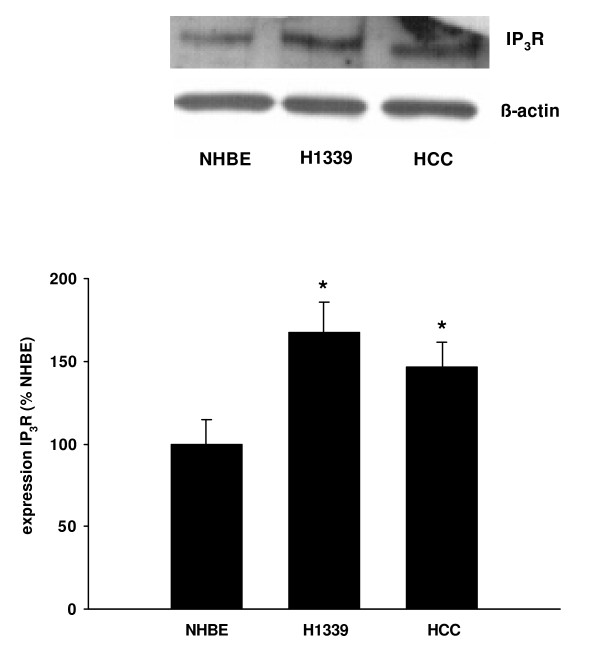
**The expression of IP_3_R was analyzed in NHBE, H1339, and HCC cells using Western Blot analysis and expressed as percentage of the IP_3_R expression in NHBE cells**. In H1339 and HCC cells, the expression of IP_3_R was increased with H1339 showing the highest expression (n = 4, * = *P *< 0.01 versus all other groups).

Ca^2+^-release channels of the ER are RyR and IP_3_R. In NHBE, H1339, and HCC cells, we found the expression RyR to be hardly detectable at all (data not shown). In contrast, IP_3_R showed substantial expression, which was higher in the lung cancer cell lines, and the highest in H1339 cells (Figure 6).

**Figure 7:**
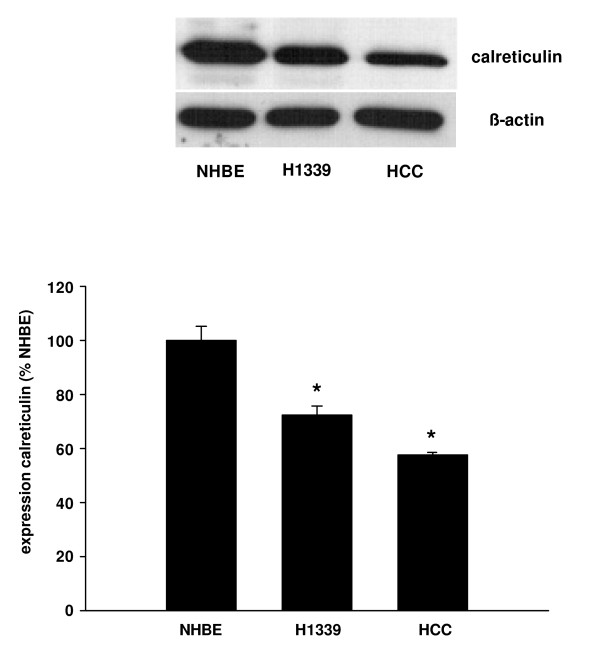
**The expression of calreticulin was analyzed in NHBE, H1339, and HCC cells using Western Blot analysis and expressed as percentage of the calreticulin expression in NHBE cells**. In H1339 and HCC cells, the expression of calreticulin was reduced with HCC cells showing the weakest expression (n = 3, * = *P *< 0.01 versus all other groups).

Within the ER, calcium is buffered by calreticulin. The expression of calreticulin was reduced in H1339 and HCC compared to NHBE cells with the lowest levels of expression being found in HCC cells (Figure 7).

**Figure 8:**
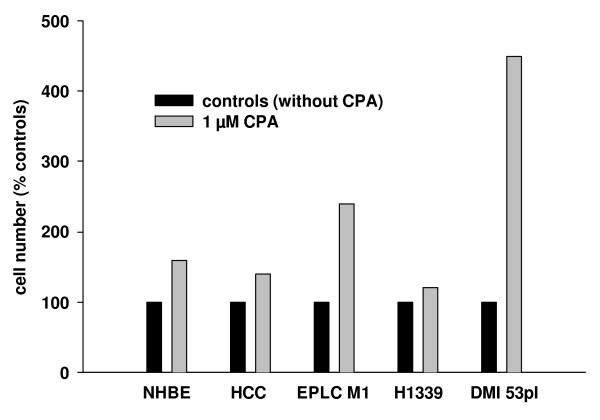
**Cells were treated with 1 μM CPA for 24 h to inhibit SERCA**. The cell number was assessed after 24 h and expressed as percent of the non-treated controls. In NHBE cells, non-small cell lung cancer cells (HCC and EPLC M1), and small cell lung cancer cells (H1339 and DMI 53 pI) the cell number was higher after CPA treatment.

In order to directly investigate the effect of a reduction of the [Ca^2+^]_ER _on the cell number, we treated the cells with CPA and assessed the cell number after 24 h. In these experiments, we used an additional non-small cell lung cancer cell line (EPLC M1, squamous cell carcinoma) and an additional small cell lung cancer cell line (DMI 53 pI). In both cell lines, the ATP-induced increase in [Ca^2+^]_C _was independent from Ca^2+^-influx from the extracellular space (data not shown). Treatment with CPA caused in NHBE cells and all lung cancer cell lines an increase in cell number compared with non-treated controls (Figure 8).

## Discussion

In this study, we showed that the contribution of Ca^2+^-influx from the extracellular space to intracellular Ca^2+^-homeostasis varied between lung cancer cell lines. However, in those cell lines in which Ca^2+^-influx played a minor role (H1339 and HCC) the ER Ca^2+^-content was reduced compared to NHBE cells. The reduced Ca^2+^-content in H1339 and HCC cells correlated with a reduced expression of SERCA 2 pumping calcium into the ER, an increased expression of IP_3_R releasing calcium from the ER, and a reduced expression of calreticulin buffering calcium within the ER. Reducing the ER Ca^2+^-content with CPA for 24 h led to an increased cell number.

The origin of the various lung carcinomas is still controversially being discussed. While squamous cell lung carcinomas are believed to origin from metaplastic bronchial epithelium, many authors believe small cell lung carcinomas to origin from neuro-epithelial bodies. But, the origin of large cell carcinomas and adeno carcinomas is less clear. However, being forced to choose a "normal" tissue to compare the malignant cell lines with, we decided to use normal human bronchial epithelial cells as a reference knowing that this choice constitutes a compromise.

SERCA is an ER transmembrane protein, consisting of a single polypeptide chain folded into four major domains, and is encoded by the genes ATP2A1-3 [5]. So far, 3 isoforms have been identified. As SERCA serves to maintain the concentration gradient between the cytoplasm and the ER by pumping calcium into the ER, SERCA has been regarded as a potential mediator of alterations of the ER Ca^2+^-content. In heart failure, the ER Ca^2+^-content of cardiac myocytes has been found to be reduced due to altered expression of SERCA [6]. In our laboratory, bronchial hyperreactivity in an asthma model was correlated with increased Ca^2+^-content in the sarcoplasmic reticulum of airway smooth muscle cells [4]. Further, in an interleukin based asthma model, the increased Ca^2+^-content was at least partially caused by increased expression of SERCA [7]. Several studies investigated the expression of SERCA in normal and tumor tissue reporting downregulation of this ATPase in cancer [8-11]. But, in colorectal cancer, Chung et al. reported that SERCA 2 mRNA was increased compared to normal tissue [12]. Moreover, increased SERCA 2 protein levels were correlated with serosal invasion, lymph node metastasis, advanced tumor stage and poorer survival-rate. Hence, an altered Ca^2+^-content of the ER might not only be involved in the early steps of carcinogenesis but may also cause further malignant transformation towards an invasive and aggressive phenotype.

Investigating the correlation of SERCA expression, [Ca^2+^]_ER _and proliferation, Legrand et al. showed that in prostate cancer cells an increased growth rate was correlated with higher [Ca^2+^]_ER _and increased SERCA 2 expression [13]. A decreased growth rate was correlated with decreased [Ca^2+^]_ER _and decreased expression of SERCA 2b. Neuroendocrine differentiation of prostate cancer cells is considered to mark increased aggressiveness of cancer growth. Vanverberghe et al. showed that neuroendocrine differentiation in these cells was associated with apoptosis resistance probably due to decreased filling of the ER Ca^2+^-store caused by under-expression of SERCA 2 and calreticulin [14]. But, Crepin at al. reported that prolactin stimulated proliferation in immortalized prostate cells through increased [Ca^2+^]_ER _due to increased SERCA 2 expression [15]. In a comprehensive review, Lipskaia proposed that proliferation is associated with a sustained increase in [Ca^2+^]_c _or sustained Ca^2+^-oscillations, decreased refilling of the ER because of SERCA inhibition, and enhanced store operated Ca^2+^-entry from the extracellular space [16]. Apparently, the relationship between [Ca^2+^]_ER_, SERCA expression and tumor growth varies between studies, cell types and differentiation status. However, an altered ER Ca^2+^-homeostasis is obviously involved in malignant transformation. To our knowledge, this is the first report showing an altered ER Ca^2+^-homeostasis in lung cancer cells.

The IP_3_R is a Ca^2+^-channel composed of 4 subunits, which releases calcium upon the binding of IP_3 _[17]. Sakakura and colleagues showed that the IP_3_R was overexpressed in a gastric cancer cell line established from peritoneal dissemination, but weakly expressed in a gastric cancer cell line established from a primary tumor as well as in normal gastric epithelial cells [18]. The authors therefore suggest a role for the IP_3_R in the transition to a metastatic phenotype. Our finding of increased IP_3_R expression in H1339 and HCC cells is in agreement with *in vivo *data obtained from patients with resectable NSCLC, where Heighway et al. found amplification of the IP_3_R gene in the tumor tissue compared to normal tissue [19].

Calreticulin is a 46-kDa chaperone that binds calcium in the lumen of the ER with high capacity [20]. It also participates in the folding of newly synthesized proteins. Recently, a role for calreticulin in immunogenic cell death has been proposed [21]. The authors reported that anthracyclines and γ-irradiation induced translocation of calreticulin to the plasma membrane thereby stimulating immunogenic cell death. In this context, our finding of reduced calreticulin expression in lung cancer cells could be of particular importance.

A decreased [Ca^2+^]_ER _is regarded as a pathophysiological mechanism in heart failure [6]. Istaroxime is a SERCA activator that has been successfully tested in a clinical phase 1–2 trial and found to be well tolerated and to improve cardiac function [22]. As substances altering the intracellular Ca^2+^-homeostasis become available for clinical use, the altered Ca^2+^-homeostasis of cancer cells may become a valuable target to improve therapeutic options in lung cancer.

## Conclusion

In our study, we showed that in H1339 and HCC cells the ER Ca^2+^-content was reduced compared to NHBE cells. The reduced Ca^2+^-content correlated with a reduced expression of SERCA 2 pumping calcium into the ER, an increased expression of IP_3_R releasing calcium from the ER, and a reduced expression of calreticulin buffering calcium within the ER. The differences in the intracellular Ca^2+^-homeostasis between lung cancer and normal bronchial epithelial cells may lay the basis for new diagnostic or therapeutical approaches.

## Competing interests

The authors declare that they have no competing interests.

## Authors' contributions

AB conceived the study, carried out experiments on the Ca^2+^-signaling and drafted the manuscript. JK carried out experiments on the Ca^2+^-signaling and Western Blot analysis. AT and RMH participated in the study design and revised the manuscript critically for important intellectual content.
